# A dynamic web-based decision aid to improve informed choice in organised breast cancer screening. A pragmatic randomised trial in Italy

**DOI:** 10.1038/s41416-020-0935-2

**Published:** 2020-06-17

**Authors:** Anna Roberto, Cinzia Colombo, Giulia Candiani, Roberto Satolli, Livia Giordano, Lina Jaramillo, Roberta Castagno, Paola Mantellini, Patrizia Falini, Eva Carnesciali, Mario Valenza, Liliana Costa, Cinzia Campari, Stefania Caroli, Roberto Cosimo Faggiano, Lorenzo Orione, Bruna Belmessieri, Vanda Marchiò, Silvia Deandrea, Anna Silvestri, Daniela Luciano, Eugenio Paci, Paola Mosconi

**Affiliations:** 1 Istituto di Ricerche Farmacologiche Mario Negri IRCCS, Milano, Italy; 2 Zadig, Agenzia di Editoria Scientifica, Milano, Italy; 3 SSD Epidemiologia e Screening - CPO Piemonte - AOU Città della Salute e della Scienza, Torino, Italy; 4 SC Screening e Prevenzione Secondaria, Istituto per lo Studio, la Prevenzione e la Rete Oncologica - ISPRO, Firenze, Italy; 5 UO Centro Gestionale Screening, ASP di Palermo, Palermo, Italy; 6 U.O.S. Screening Mammografico, ASP di Palermo, Palermo, Italy; 7 S.S. Screening Oncologici - Azienda Unità Sanitaria Locale – IRCCS di Reggio Emilia, Reggio Emilia, Italy; 8 Centro Screening Cuneo, ASL CN1, Cuneo, Italy; 9 UOC Medicina Preventiva delle Comunità – Screening, ATS della Città Metropolitana di Milano, Milano, Italy; 10 Lega Italiana per la Lotta contro i Tumori, Sezione Firenze, Italy

**Keywords:** Education, Cancer

## Abstract

**Background:**

Improving the quality of information and communication is a priority in organised breast cancer screening and an ethical duty. Programmes must offer the information each woman is looking for, promoting informed decision-making. This study aimed to develop and evaluate a web-based dynamic decision aid (DA).

**Methods:**

A pragmatic randomised trial carried out in six regional organised screening programmes recruited women at the first invitation receiving DA or a web-based standard brochure (SB). The primary outcome was informed choice measured on knowledge, attitudes, and intentions. Follow-up period: 7–10 days. Secondary outcomes included participation rate, satisfaction, decisional conflict, and acceptability of DA.

**Results:**

Two thousand one hundred and nineteen women were randomised and 1001 completed the study. Respectively, 43.9% and 36.9% in the DA and SB reached the informed choice. The DA gave a 13-point higher proportion of women aware about overdiagnosis compared to SB (38.3% versus 25.2%, *p* < 0.0001). The percentage of women attending screening was the same: 84% versus 83%. Decisional conflict was significantly lower in the DA group (14.4%) than in the SB group (19.3%).

**Conclusion:**

DA increases informed choice. Complete information including the pros, cons, controversies, and overdiagnosis–overtreatment issues boost a woman’s knowledge without reducing the rate of actual screening participation.

**Clinical trial registration:**

ClinicalTrials.gov number NCT 03097653.

## Background

Breast cancer (BC) is the leading cancer site among European women, with about 400,000 new cases every year. The incidence of BC has continued to rise in almost all European countries in recent decades, while mortality rates have fallen in many countries, partly thanks to organised BC screening programmes.^1^ According with European Code against cancer,^2,3^ in Europe, BC screening programmes, under national health services, offer mammography every 2 years mainly to women aged 50–70 years, with monitoring of performance and quality.^4^ In Italy in 2015/2016, about six million women were invited and about 60% attended; recently in some regions, invitations were extended to 45–49- and 70–74-year-old women.^5,6^

Quality of information and communication and promotion of individual decision-making on mammography screening based on knowledge of its benefits and harms represent a priority.^7^ Information and campaigns about the benefits and harms of cancer screening have proved insufficient in many surveys, which have evaluated the contents of the leaflets or websites on women’s knowledge. Most of them stress the benefits more than harms; false positive results and overdiagnosis–overtreatment, without quantitative estimates, are occasionally reported.^8–10^ Some studies have shown that many women confused early diagnosis with prevention, misunderstanding the value of screening.^11,12^

Information on mammography screening is a complex issue partly because of the scientific controversies on the evidence and quantification of the benefit and harm ratio, since a 2001 Cochrane Systematic review denied the impact on mortality of mammography screening and estimated high risks of overdiagnosis–overtreatment.^13^ In 2010, European screening researchers assessed the published outcomes in terms of benefits and harms of the ongoing and oldest screening programmes.^14^ In the same year, the Independent UK Panel confirmed the evaluation of the efficacy of mammography screening.^15^ Nevertheless, there is still strong pressure for ending the mammography screening programmes^15^; in Switzerland in 2014 and in France in 2016, review and consultation reports have opened the debate again on the utility of BC screening programmes.^16^ An IARC monograph in 2002, updated in 2015, confirmed the validity of screening programmes,^17^ as well the new European guidelines.^18^ In 2013, the Cochrane systematic review was updated confirming the initial evaluation.^19^ However, this sharp debate has not put an end to the controversy.

Each woman should decide on the basis of experience and personal values, despite the individual invitation by the organised screening programmes. A decision aid (DA) that explains benefits and harms of screening better than the “regular” invitation letter is suggested by the European recommendations,^18^ according with a Cochrane review on DAs in screening decisions.^20^ DAs improve patients’ knowledge, clarify values, reduce decisional conflict and encourage women to take a more active role in decision-making without anxiety.^20^

The Donnainformata-Mammografia project (informed women mammography) was designed to develop and evaluate a web-based dynamic DA in a pragmatic randomised trial, i.e. as part of organised mammography screening programmes’ practice.^21^ The aim was to compare DA with a web-based standard static brochure (SB).

## Methods

Six regional organised screening programmes invited women eligible for a first invitation—aged >50 years in 4 screening programmes and aged >45 years in 2, without personal history of BC—with an official letter sent about 30 days before the screening invitation. According to the protocol published elsewhere,^21^ three programmes participated since the beginning, three additional programmes joined the study later due to the difficulties to achieve the required sample size.

### Development of the DA and SB

The contents and layout of the DA were developed after a literature review^17,22–24^, a collection of screening information materials, a focus group phase including 18 women, and 4 in-depth semi-structured interviews (data not reported). We included women aged 45–54 years who had participated in a screening programme in the previous 6 months not having a positive result. Women with a personal or family history of BC were excluded. The aim was to gather information needs, knowledge and attitudes towards BC screening and comments on a draft of the home page of the DA including the main topics covered in the tool. A convenience sample of these women was further interviewed to provide feedback on pre-final version of the DA; findings guided the wording, the contents and the layout.

Scientific evidence was based on the Euroscreen^14^ and Independent UK Panel.^25^ Development of DA started from the criteria of the International Patient Decision Aid Standards Collaboration.^26^

In the homepage of the DA, a nudging-like approach was used to highlight four main sections: What is BC? What is mammography screening? What are its benefits and harms? What results can be expected from mammography screening? Women were free to decide which sections to access and to move to other pages linked from the homepage.

Topics were described in plain language and the benefit/harm balance was defined on the basis of literature.^14,15,25^ The information covered controversial issues such as false positive results and overdiagnosis–overtreatment (www.donnainformata-mammografia.it).

The SB was assembled on the basis of brochures used in the participant-organised screening programmes of Turin, Florence and Palermo (Supplementary File 1). Comparative information regarding the DA and SB is reported in Table 1. DA provided a module listing issues and concerns that can affect screening decision, and each woman was asked to state the importance of each item (Supplementary File 2).

**Table 1:** Comparison of the decision aid and standard brochure.

	Decision aid	Standard brochure
Portrayal	Online decision aid, 19 screens, each covering one topic, not printable	Online static page divided into four sections, printable
Visual aspects	Short coloured text, graphics and pictures, bullet points, hyperlink	Black and white text, no graphics or pictures
Language and contents	Plain language. Contents are defined on the basis of the literature and guidelines	Plain language. Contents combine the best information from three leaflets for organised screening programmes
Key contents	What is mammography screening? The pros and cons of mammography screening. What might happen in the next 30 years? At what age is mammography screening recommended? The risks related to radiation. Organised mammography screening programme, a quality programme? What result will the mammography give? What happens at each screening? Diagnostic programmes in uncertain cases. Breast density. What is breast cancer and how can it be treated? Differences between false positives and overdiagnosis. The balance between benefits and harms. How are the rates of specific mortality reduction and overdiagnosis measured? Different estimates of the reduction of mortality due to breast cancer. Different overdiagnosis estimates	What is mammography? –Why do a mammography? –The limits of mammography. –What result will the mammography give?
Quantitative data	Quantitative data from the UK Panel^25^ and the Euroscreen studies.^14^ Absolute numbers reported in the text for: –positive and negative screening results –false positive and negative –overdiagnosis –mortality with screening –mortality without screening Comparative data on X-ray exposure	Absolute numbers in the text for: –false positive cases –overdiagnosis –mortality with screening
Controversy and disagreement on quantification of harms and benefits	Quantitative estimates from Cochrane Review^19^	Not mentioned
In-depth information	Reference available	None
Information on prevention	One screen on risk and protective factors with a table comparing things to do and not to do	None
Value clarification exercise	Interactive personal page with aspects leading the choice to participate in mammography screening such as values, experience, and perception of the risk of developing BC. For each aspect, it can be moved with a cursor against or in favour of participation. The whole page can be downloaded and printed	None

Any time, navigating the DA or SB, a woman could decide if she had enough information and leave the platform.

### Assessment of the impact of the DA

Women were selected from the computerised demographic list of the screening programmes. Each eligible woman received an invitation to participate and a personal code number to log into the platform. After signing the informed consent form and compiling a baseline questionnaire, women were randomised to the DA or SB in a 1:1 ratio. After 7–10 days, women were contacted via email or short message service and invited to complete a follow-up questionnaire. Every 3 days, a reminder was sent to non-responders until the scheduled mammography date.

To define the outcome, we start from a Cochrane review,^20^ and we reviewed literature including randomised controlled trials on BC screening DAs published from 2012 to 2016.

The primary outcome was informed choice, assessed as a dichotomous variable: a woman with adequate knowledge (>8/13 correct answers) and consistent attitude and intention was considered as expressing informed choice.^22–24,27^ Secondary outcomes were participation rate, satisfaction on information, decisional conflict,^28^ time spent on the platform and the acceptability of DA (Supplementary File 3).

### Statistical analysis

A sample of 816 women was required (5% alpha, 80% power).^21^ For the primary analysis, done on an intention-to-treat basis in accordance with the protocol, all the women randomised, compliant to follow-up, were included in the final analysis.^21^ The proportions of women were compared with a chi-square test (two-sided, *P* < 0.05). A *t* test was used for continuous endpoints (two sided, *P* < 0.05). Analyses were done with the SAS statistical software, version 9.4.

## Results

**Fig. 1:**
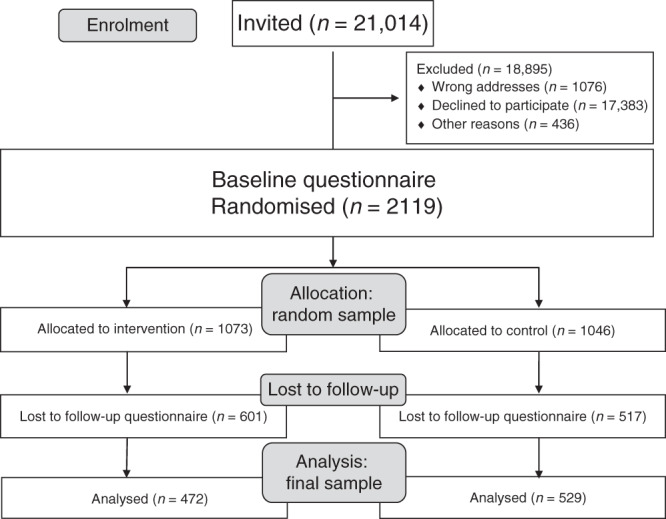
Consort flow diagram: overview of trial participation.

A total of 21,014 women were invited (Fig. 1), with a recruitment phase lasting 15 months, from September 2017 to December 2018. Among the organised BC screening programmes, the number of women contacted ranged from 1655 to 7278. Finally, 2119 women signed the informed consent form, completed the baseline questionnaire and were randomised (randomised sample). Of these, 1001 completed the follow-up questionnaire (final sample).

Table 2 illustrates the characteristics of women; there was no significant imbalance. Most women had an Italian citizenship, were married, employed and used internet sporadically for health information. About two-thirds had relatives and/or acquaintances who had received a diagnosis of BC, about 70% had a previous mammography and most (70%) perceived having a BC risk in line with women of the same age.

**Table 2:** Main characteristics of the women involved in the study.

	Randomised sample		Final sample	
	Decision aid, *N* = 601	Standard brochure, *N* = 517	Decision aid, *N* = 472	Standard brochure, *N* = 529
	*N* (%)	*N* (%)	*N* (%)	*N* (%)
Age (in years), mean (SD)	49.7 (3.1)	49.7 (3.3)	49.0 (3.0)	49.2 (3.3)
Education				
Elementary	9 (1.5)	8 (1.6)	2 (0.4)	4 (0.8)
Lower middle	103 (17.2)	109 (21.2)	69 (14.7)	71 (13.5)
Higher middle	297 (49.5)	229 (44.5)	234 (49.8)	272 (51.7)
Degree	179 (29.8)	157 (30.5)	156 (33.2)	170 (32.3)
Other	12 (2.0)	12 (2.3)	9 (1.9)	9 (1.5)
Nationality				
Italian	579 (96.5)	498 (96.7)	458 (97.4)	506 (96.2)
Marital status				
Married or cohabitant	432 (72.1)	370 (71.8)	342 (72.8)	386 (73.4)
Employment status				
Paid work	466 (77.8)	405 (78.6)	366 (77.9)	425 (80.8)
Use internet for health info				
Never	160 (26.7)	136 (26.4)	128 (27.2)	111 (21.0)
A few times/month	289 (48.2)	260 (50.5)	230 (48.9)	271 (51.5)
At least once/week	54 (9.0)	48 (9.3)	49 (10.4)	44 (8.4)
Several times/week	37 (6.2)	28 (5.4)	29 (6.2)	43 (8.2)
Daily				
Acquaintance/family with BC (yes)	396 (66.0)	347 (67.4)	331 (70.4)	380 (72.2)
Previous tumours (yes)	43 (7.2)	32 (6.2)	24 (5.1)	38 (7.3)
Perceived risk of BC				
Much lower	26 (4.4)	24 (4.7)	24 (5.1)	17 (3.2)
A bit lower	47 (7.9)	38 (7.4)	23 (4.9)	45 (8.6)
About the same as average women	434 (72.6)	373 (72.6)	347 (74.0)	381 (72.6)
A bit higher	75 (12.5)	61 (11.9)	61 (13.0)	67 (12.8)
Much higher	16 (2.7)	18 (3.5)	14 (3.0)	15 (2.9)
Previous mammography (yes)	430 (71.5)	355 (68.7)	311 (65.9)	372 (70.3)
Participation in faecal occult blood test screening (yes)	101 (16.9)	93 (18.1)	84 (17.9)	106 (20.2)
Participation in Pap test screening (yes)	462 (77.1)	418 (81.2)	357 (76.0)	410 (78.2)

Some differences are due to missing data.

**Fig. 2:**
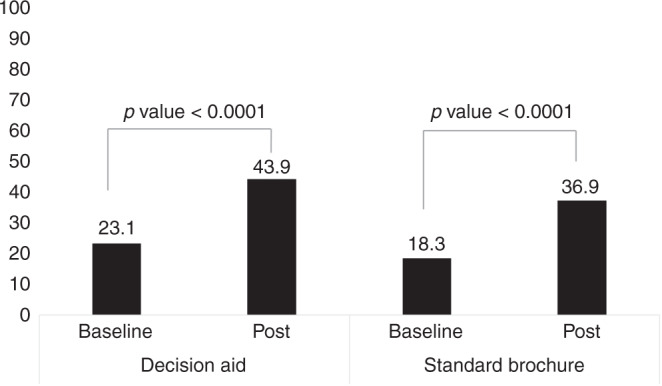
Informed choice: main outcome of the study.

Figure 2 shows the proportion who reached the informed choice: 43.9% (207/472) and 36.9% (195/529) in the DA and SB arms, respectively (*P* = 0.0328). At the baseline questionnaire, 23.1% in the DA and 18.3% in the SB group (*P* = 0.0694) had adequate knowledge and consistent attitude and intention, meaning an increase in informed choice of 20.8% versus 18.6%, respectively, in the DA and SB arms.

Details of the primary outcome shows that information about screening and benefits was generally good, whereas only about 65% recognised the possibility of a false negative result (interval cancer). The DA gave a 13-point higher proportion of women aware about overdiagnosis compared to the SB (38.3% versus 25.2%, *P* < 0.0001). Overtreatment had similar frequencies (37.7% versus 26.6%, *P* = 0.0002). Information about controversies in mammography screening was poor in the SB arm where this information was not given and limited in the DA (11.0% versus 27.2%, *P* < 0.0001). On numerical items, the DA women had a worse idea of the frequency of BC mortality for women in the same age group, both with and without mammography screening programmes (DA 21.1% versus SB 25.6%, *P* = 0.0944). The SB group responded more correctly about the estimates of overdiagnosis (DA 19.6% versus SB 26.4, *P* = 0.0117). Attitude and intention towards BC screening were high in both the groups (>90%; Table 3).

**Table 3:** Details of primary outcome.

	Decision aid *n* = 472	Standard brochure *n* = 529	*P*-value
	*n* (%)	*n* (%)	
Knowledge conceptual items, right answers			
1. Screening is a mammography you have when you're healthy	454 (97.4)	497 (96.0)	0.1982
2. An organized mammography screening program can detect a breast cancer in an early stage and lead to less invasive surgery and treatment	460 (98.7)	511 (98.5)	0.7368
3. Regular mammography every two years in women who are well does not prevent the risk of BC	50 (10.7)	56 (10.8)	0.9664
4. Women who do not have screening mammography is more likely to die from BC	444 (95.3)	492 (94.8)	0.7287
5. A screening mammography does not find every BC	299 (64.2)	341 (65.7)	0.6129
6. Not all the women with an abnormal screening mammography result have BC	463 (99.4)	512 (98.7)	0.2705
7. Overdiagnosis means that screening finds a BC that would never have caused trouble	179 (38.3)	131 (25.2)	**<0.0001**
8. Screening leads some women with a harmless cancer to get treatment they do not need (true)	177 (37.7)	138 (26.6)	0.0002
9. The organized mammography screening program, the presence of two expert radiologists increases the ability to identify a BC	469 (100)	519 (100)	-
10. The usefulness of an organized mammography screening program is questioned by some doctors and researchers	126 (27.2)	57 (11.0)	<**0.0001**
Knowledge numerical items, right answers For the next few questions, I would like you to imagine 1000 ordinary women who are 50 years old who have participated regularly in organized mammography screening program for 30 years…			
1. How many women do you think will avoid dying from BC because of screening?	92 (19.6)	137 (26.4)	**0.0117**
2. How many women do you think will be diagnosed and treated for a BC that is not harmful?	300 (64.0)	361 (69.7)	0.0562
3. Now, I would like you to imagine 1000 ordinary women who are 50 years old who have not participated in organized mammography screening program, in their next 30 years…. How many die of BC?	99 (21.1)	133 (25.6)	0.0944
Attitude toward BC screening			
Positive	432 (91.5)	489 (92.4)	0.0922
Intentions toward BC screening			
Positive	461 (98.7)	502 (97.8)	0.0230

Some differences are due to missing data

Statistically significant *P* values are in bold.

Regarding secondary outcomes (Table 4), a large proportion of women had no decisional conflict, and it was significantly lower in the DA group (14.4%) than in the SB group (19.3%) (*P* = 0.0403). In both the groups, most women (>90%) considered the information as enough and clear, and they would recommend it to other women. The information conveyed was mainly considered in favour of BC screening (around 65% in both the groups) and useful for deciding (around 70% in both the groups). Some information regarding benefits was considered new. The percentage of women attending BC screening was the same in the two groups: 84% of women surfing the DA versus 83% for women receiving the SB (*P* = 0.6537). On average, 4.8 (SD 4.2) pages were visited on the DA; women spent more time on the pages “The main risk and protective factors” and “How are the rates of specific mortality reduction and overdiagnosis measured?”. The median time spent, considering a mean of 215 words per screen, ranged between 97 and 16 s. According to the number of women who visited each page, besides the homepage, the most visited pages were related to pro and cons, quality and results of BC screening programmes; what happens at each round of screening; what is BC and related treatment; and the balance between benefits and harms of BC screening.

**Table 4:** Secondary outcomes.

	Decision aid, *N* = 472	Standard brochure, *N* = 529	*P* value
	*N* (%)	*N* (%)	
Participation in BC screening (yes)	376 (84.1)	416 (83.0)	0.6537
Satisfaction with information			
Was there enough information			**0.0162**
Too much	17 (3.6)	6 (1.2)	
Too little	19 (4.1)	31 (6.0)	
Fair	432 (92.3)	480 (92.8)	
Was the information on benefit new to you?			0.1000
All or almost all	32 (6.8)	53 (10.3)	
Some	337 (71.9)	345 (66.7)	
None	100 (21.3)	119 (23.0)	
Was the information on harm new to you?			0.0671
All or almost all	49 (10.5)	51 (9.9)	
Some	288 (61.4)	285 (55.1)	
None	132 (28.1)	181 (35.0)	
Was the information clear?			0.4759
All or almost all	434 (92.5)	472 (91.3)	
Some	35 (7.5)	45 (8.7)	
The information seemed…			0.3702
In favour of screening	296 (63.1)	342 (66.1)	
Balanced	173 (36.9)	174 (33.7)	
Against screening		1 (0.2)	
Did it help you to decide?			0.8499
Yes	330 (70.4)	360 (69.6)	
Not much	108 (23.0)	118 (22.8)	
No	31 (6.6)	39 (7.5)	
Would you recommend it to other women?			0.2129
Yes	454 (96.8)	507 (98.1)	
Not much	15 (3.2)	9 (1.7)	
No		1 (0.2)	
Was the controversy new to you?			
All or almost all	70 (15.0)		
Some	338 (72.0)		
None	61 (13.0)		
Decisional conflict			
Decisional conflict (score ≥3)	68 (14.4)	102 (19.3)	**0.0403**
No decisional conflict	404 (85.6)	427 (80.7)	
Median time spent, s			
Home page	42		
Mammography screening	22		
Pros/cons of mammography screening	55		
What happens in the next 30 years?	56		
Age recommended	30		
Radiation risks	31		
Doses of radiation from different examinations	41		
Organised mammography screening programme, a quality programme	41		
Result of mammography	36		
What happens at each screening?	50		
Diagnostic programmes in uncertain cases	25		
Breast density	37		
Breast cancer and treatment	46		
Risk/protective factors	97		
Differences in false positives/overdiagnosis	30		
Balance between benefits/harms	34		
Rates of specific mortality reduction and overdiagnosis	16		
Different estimates of the reduction of BC mortality	35		
Different overdiagnosis estimates	46		

Statistically significant *P* values are in bold.

According to the value each woman gave to the items in their decision-making process, cancer mortality, breast conservation, risk of developing a BC and quality of the organised screening programmes are the main factors judged as important in the decision to participate (or not) in mammography screening. Harms and controversies seem not to influence this choice (Supplementary File 4, Fig. 1).

## Discussion

This pragmatic trial shows that the DA increases informed choice compared to the SB, without reducing the rate of screening participation. The attitude towards screening and intention to participate were extremely positive at baseline—>90%—and did not change at follow-up assessment. Most women had already had a mammography and were unaware of the current controversies. These findings are in line with the focus groups held at the beginning of the project showing a positive attitude towards screening.

The DA offered detailed qualitative and quantitative information on benefits, harms and controversies, which are rarely presented in the standard materials received by women. We provided these topics at the same time making explicit our position towards the national health BC screening programme, to be transparent and consistent with our position as promoters.

At baseline, one-third of the women knew about overdiagnosis, whereas <15% knew the meaning of overtreatment. A higher frequency of women in the DA group correctly responded on overdiagnosis–overtreatment. However, the proportion was low compared to the proportion of women responding correctly (both in DA and SB) to the false positive question. As seen during the focus groups, overdiagnosis–overtreatment issues are complex, sometimes obscure and not intuitive—as shown in other studies too.^29,30^

More women responded correctly to the false positive question than to the false negative one (both in DA and in SB). This is in line with the findings by Perez-La Casta^31^ in the InforMa trial conducted in a similar screening programme setting in Spain. Considering other studies on DAs and perception of women towards screenings,^32^ the risk of false positive results is known by women and underlined by information conveyed to women more often than the false negative one. The psychological impact of a false positive result can be severe for a woman, compared to a false negative result that is difficult to define on a single basis. Furthermore, in BC screening the false negative rate is lower than the false positive one.

The numerical items had a lower frequency of correct responses than conceptual items; however, the numerical item on overdiagnosis–overtreatment had correct responses from 64.0% in the DA group and 69.7% in the SB group. The SB presented fewer numerical information than the DA and was accessible on one printable page. This may have facilitated a correct answer.

From the baseline in both the groups, there was a significant increase in the proportion of women’s knowledge, which was the dimension that mainly influenced the informed choice. It might also indicate that the SB, a mix of brochures used in three BC screening programmes, is a potentially useful tool to improve knowledge and may be more informative than the leaflets of the different Italian screening programmes. The DA and SB reported comparable information with different details according to the ethical duty to convey the same standard of information that women generally receive within screening programmes.

The time spent surfing the DA was limited though each section was short (77–416 words). The level of satisfaction with the information was high in both arms, hitting our goal to produce material matching the interest in being informed and our aim to present complex information in a straightforward, user-friendly way.

Women perceived the DA and SB as being in favour of BC screening, despite the fact that the DA illustrated pros and cons, overdiagnosis–overtreatment and presented different ways to assess the benefit–harm ratio (controversies). This might reflect the setting in which the study was conducted—i.e. within screening programmes.

Women receiving the DA reported less decisional conflict than the SB group; they felt better supported and advised about their choice and more confident about it.

These results match the findings of reviews showing that DA about BC screening can improve knowledge and promote informed decision-making.^33^ Recent clinical trials also showed that complete information positively influences the informed choice.

Hersch et al.^22^ recruited women aged 48–50 years by telephone in a community-based trial to compare two kinds of DA, differing in including or not information on overdiagnosis–overtreatment. The comparison group received a high standard of information, more than usual leaflets. The authors concluded that information on overdiagnosis increased the number of women making an informed choice and that significantly fewer women expressed positive attitudes towards screening.

Reder and Kolip^34^ compared an SB with a new DA in 50-year-old women invited to participate in a BC organised screening programmes. Both groups had a very positive attitude towards screening. The results suggested that the DA increased the level of informed choice and knowledge and reduced decisional conflict.

Perez-La Casta^31^ in the InforMa trial assessed the effect of receiving information about benefits and harms of BC screening on informed choice in women likely to be invited in organised programmes. Spanish 50-year-old women were randomly selected and allocated to a DA or an SB where harms were not mentioned. Similarly to our study, about 80% of the Spanish participants had had a previous mammogram. The study shows a strong increase in the number of women who made an informed choice. Intention to be screened was high in both the groups with no difference in screening participation.

It is hard to compare the results of these studies because of different control leaflets used, differences in how the primary outcome was measured and in setting, cultural environment, type of information conveyed, and control arm. However, increase in knowledge and informed choice always emerged.

Like the InforMa^31^ and Reder^34^ studies, our study was part of organised BC screening programmes, facilitating the adoption of the DA in standard practice, in addition to well-balanced informative leaflets, as the one developed within our study.

Being able to conduct this pragmatic trial in the context of the service screening is an important achievement.^35^ Procedures were integrated in the invitation system, contacting women in a user- friendly way and not overloading the staff involved. Organised screening has high potential for complex research investigations, facilitating informed consent and respecting women’s privacy. Outside a research context since the registration on the platform and answer to questionnaires will no longer be necessary, the DA use could be more feasible, at least in the younger age groups (45–54 years).

The DA and SB arms were balanced according to the randomisation design, the characteristics of final sample remaining balanced even considering the dropouts.

The information in the DA was iteratively revised on the basis of the best evidence available by a multidisciplinary group including experts in communicating health issues, experts with decisional tools, BC screening multi-professional scientific societies, epidemiologists and representatives of consumers and patients.

This study has several limitations. First, considering the sample size, the response rate was lower than expected, and there were about 50% dropouts. We invited more women than expected (21,014 versus 8160). This high proportion of dropouts could be also due to scarce attention of women to comply with this type of study, suggesting the need for careful review of the recruitment process. Participating women had a high level of education, which limits the generalisability of findings, in agreement with other studies.^22,31,34^ Most of the participants had already had a mammography before the invitation to the organised screening programme. This suggests that many had already received information that could have fostered the attitude and intention reported in this study. Finally, in order to participate, women had to have basic information technology skills. It is likely that technical developments will offer more user-friendly tools for sharing information, increasing users’ knowledge and facilitating decision-making in complex healthcare areas, such as mammography screening. However, to reduce the imbalances between population subgroups, it will be essential to identify alternative strategies and means to effectively reach women who do not use computers. As recently reported in the guidelines of the European commission, DAs are recommended taking account of the cultural contexts and literacy levels.^18^

Experience in the construction of a flexible, simple and accurate DA offers a valuable opportunity to reach many women. This will be possible in the near future using smartphones, and work in that direction is needed.

Complete information including the pros, cons, controversies and overdiagnosis–overtreatment issues boost a woman’s knowledge without her losing interest in BC screening. Attitudes towards screening are very positive, and women do not change them because of knowledge of the benefit–harm ratio when mammography screening is proposed as an evidence-based, advantageous practice organised in the framework of the National Health Service.

Our findings, in line with a general consensus on the right to have access to correct information,^7^ confirm that a screening service has the duty to provide the best possible information about benefit and harms of screening to facilitate decision-making and ensure informed consent by women.^36^

## Supplementary information

Supplementary files

## Data Availability

Data supporting the results will be provided on public websites or archives.
