# Correction: A dynamic web-based decision aid to improve informed choice in organised breast cancer screening. A pragmatic randomized trial in Italy

**DOI:** 10.1038/s41416-021-01437-3

**Published:** 2021-05-11

**Authors:** Anna Roberto, Cinzia Colombo, Giulia Candiani, Roberto Satolli, Livia Giordano, Lina Jaramillo, Roberta Castagno, Paola Mantellini, Patrizia Falini, Eva Carnesciali, Mario Valenza, Liliana Costa, Cinzia Campari, Stefania Caroli, Roberto Cosimo Faggiano, Lorenzo Orione, Bruna Belmessieri, Vanda Marchiò, Silvia Deandrea, Anna Silvestri, Daniela Luciano, Eugenio Paci, Paola Mosconi

**Affiliations:** 1 Istituto di Ricerche Farmacologiche Mario Negri IRCCS, Milano, Italy; 2 Zadig, Agenzia di Editoria Scientifica, Milano, Italy; 3 SSD Epidemiologia e Screening - CPO Piemonte – AOU Città della Salute e della Scienza, Torino, Italy; 4 SC Screening e Prevenzione Secondaria, Istituto per lo Studio, la Prevenzione e la Rete Oncologica - ISPRO, Firenze, Italy; 5 UO Centro Gestionale Screening, ASP di Palermo, Palermo, Italy; 6 U.O.S. Screening Mammografico, ASP di Palermo, Palermo, Italy; 7 S.S. Screening Oncologici - Azienda Unità Sanitaria Locale – IRCCS di Reggio Emilia, Reggio Emilia, Italy; 8 Centro Screening Cuneo, ASL CN1 Cuneo, Italy; 9 UOC Medicina Preventiva delle Comunità – Screening, ATS della Città Metropolitana di Milano, Milano, Italy; 10 Lega Italiana per la Lotta contro i Tumori, Sezione Firenze, Italy

**Keywords:** Education, Cancer

Correction to: *British Journal of Cancer* 10.1038/s41416-020-0935-2, published online 17 June 2021

The original version of this article unfortunately contained a mistake in Table 3. Since the publication of the article the authors found a mistake in the label of one of the variables in Table 3. This mismatch results in incorrect listing of numbers and percentages. The correct table can be found below. The authors apologise for the mistake. The original article has been corrected.

**Table 3:** Details of primary outcome.

	Decision aid *n* = 472	Standard brochure *n* = 529	*p*-value
	*n* (%)	*n* (%)	
**Knowledge conceptual items, right answers**			
1. Screening is a mammography you have when you’re healthy	454 (97.4)	497 (96.0)	*0.1982*
2. An organized mammography screening program can detect a breast cancer in an early stage and lead to less invasive surgery and treatment	460 (98.7)	511 (98.5)	*0.7368*
3. Regular mammography every two years in women who are well does not prevent the risk of BC	50 (10.7)	56 (10.8)	*0.9664*
4. Women who do not have screening mammography is more likely to die from BC	444 (95.3)	492 (94.8)	*0.7287*
5. A screening mammography does not find every BC	299 (64.2)	341 (65.7)	*0.6129*
6. Not all the women with an abnormal screening mammography result have BC	463 (99.4)	512 (98.7)	*0.2705*
7. Overdiagnosis means that screening finds a BC that would never have caused trouble	179 (38.3)	131 (25.2)	***<.0001***
8. Screening leads some women with a harmless cancer to get treatment they do not need (true)	177 (37.7)	138 (26.6)	*0.0002*
9. The organized mammography screening program, the presence of two expert radiologists increases the ability to identify a BC	469 (100)	519 (100)	*–*
10. The usefulness of an organized mammography screening program is questioned by some doctors and researchers	126 (27.2)	57 (11.0)	***<.0001***
**Knowledge numerical items, right answers** For the next few questions, I would like you to imagine 1000 ordinary women who are 50 years old who have participated regularly in organized mammography screening program for 30 years…			
1. How many women do you think will avoid dying from BC because of screening?	92 (19.6)	137 (26.4)	***0.0117***
2. How many women do you think will be diagnosed and treated for a BC that is not harmful?	300 (64.0)	361 (69.7)	*0.0562*
3. Now, I would like you to imagine 1000 ordinary women who are 50 years old who have not participated in organized mammography screening program, in their next 30 years…. How many die of BC?	99 (21.1)	133 (25.6)	*0.0944*
**Attitude toward BC screening**			
Positive	432 (91.5)	489 (92.4)	*0.0922*
**Intentions toward BC screening**			
Positive	461 (98.7)	502 (97.8)	*0.0230*

Some differences are due to missing data.

