# Supplementary material for: A dynamic web-based decision aid to improve informed choice in organised breast cancer screening. A pragmatic randomised trial in Italy
Source: Br J Cancer. 2020 Jun 17;123(5):714–21. doi: 10.1038/s41416-020-0935-2 (PMC7462858; doi:10.1038/s41416-020-0935-2)

**Supplementary files:**

**Supplementary 1. Standard Brochure – English version**

Screening is a public health program where healthy population, at an age judged to be at risk, is invited by local health system to undergo free preventive examinations, such as mammography, Pap test or fecal occult blood test.

Screening may help anticipate cancer diagnosis and treatment, allowing less invasive surgical interventions. For breast cancer screening every woman, between 50 and 69 years of age, receives a letter of invitation with a fixed appointment to carry out a free mammography every 2 years. Scientific research has proven that screening for breast, uterus, and colorectal cancers can save many lives.

**MAMMOGRAPHY**

Currently, for women aged between 50 and 69, mammography is the most effective test to detect tumors that are not yet symptomatic and are not palpable.

It is a radiograph of the breast that uses very low X-ray doses.

To obtain a clear result it is necessary to compress the breasts. This compression can be **bothersome** or may cause slightly pain, but lasts a few seconds. The radiographer is highly qualified and the equipment used is technologically advanced and controlled. All this makes it possible to obtain images of excellent quality.

For greater security on the diagnosis, the images are examined separately by two radiologists to ensure greater accuracy. However, like all tests, it is not foolproof: in some cases, it may not recognize a tumor that is there or suspect a non-existent tumor.

**WHY A MAMMOGRAPHY?**

In Italy, breast cancer is the first cancer among women due to incidence, i.e. number of new cases of breast cancer in a year. It is the most common cancer among women, with tens of thousands new cases and 11,000 deaths. The mammography performed regularly allows an early diagnosis, before the onset of symptoms, and the possibility of intervening with a high probability of definitive recovery. Data support that, for every 1000 women aged between 50 and 69 years who regularly perform mammography, 7-9 lives are saved within 20 years.

**WHAT ARE THE LIMITS OF THE MAMMOGRAPHY?**

■ In general, every 100 women who had a mammography, about 5 must repeat the exam or undergo in-depth examinations. For 4 of them it is actually a "false alarms", as the in-depth analyses exclude the presence of a tumor (the so-called false positives). The disadvantage is mainly represented by the anxiety that a woman can experience in such situation.

■ Mammography is not always able to detect the tumor when it is present. In some cases the breast is very dense (i.e. it contains many glands) and this makes it difficult to detect the tumor (the so-called false negatives).

■ In some cases the disease develops very quickly in the interval between two screening tests (so-called interval cancers). Even if the mammography did not show anomalies, it is important to pay attention to breast changes such as hardening, skin deformation, leakage of fluid from the nipple and the presence of axillary nodules. If you note such anomalies, you should contact your doctor immediately.

■ Some anomalies, among those detected by mammography, are not destined to become invasive tumors and to compromise the woman's health (the so-called overdiagnosis).

In these cases (1 case of cancer every 10 diagnosed) it is possible to have not necessary interventions. Unfortunately, this is inevitable as it is still not possible to distinguish from the beginning life-threatening tumors from the others.

**WHAT CAN THE OUTCOME OF THE MAMMOGRAPHY BE?**

■ If nothing suspicious appears, you will receive a negative result letter.

It is important to know that the purpose of mammographic screening is to identify malignant tumors recognizable by x-rays; other breast diseases are not considered.

■ If mammography shows doubtful images, you will be contacted and invited to go to the screening center to carry out in-depth examinations, which can include other mammography, echography or a collection of a small sample of breast tissue (biopsy). Most of these doubt cases are not due to the presence of a tumor, nevertheless it is necessary to perform these additional exams to be sure.

**Supplementary 2. Decision-aid – English version**

**Home-page**

Dear Miss, thank you for having agreed to help us.

In a few weeks, you will receive an invitation to take part in an organized mammography screening program. According to the Italian Ministry of Health recommendations, based on scientific studies, the use of this test in the screening program can reduce mortality due to breast cancer among women of your age.

Obviously, the decision to participate or not is entirely up to you.

Here you will find some up-to-date information about mammography screening, its pros and cons, including the controversies and different opinions of experts.

We know that your choice will not be based only on this information but other aspects too will affect the decision: your life experience, your perception of the risk of developing this disease and your own values. These are very important aspects that drive many of our choices: they shall therefore recall at the end of the navigation when you make your final decision.

Before taking a decision, explore these pages, in your own time: the icon at the top “Where I am” will help you navigate When you feel you know enough, just click on “Ready to choose” at the bottom of the page.

**What is mammography screening?**

Mammography is a radiological breast examination that uses very low X-ray doses.

Mammography can identify lesions or nodes you cannot yet feel. This is why it is used for screening in women who have no symptoms or signs, so as to detect any breast cancers at an early stage.

It is advisable to take part in an organized mammography screening program because it is reliable, with quality control and a standard care schedule already sets up in case further examinations are required. Outside organized mammography screening programs there is no regular quality control by third parties, so quality is not guaranteed.

The mammography screening and any subsequent diagnosis and treatment (if necessary) within the organized mammography screening program are free of charge.

**The pros and cons of mammography screening**

Mammography screening can provide both benefits and harms, like any other medical examination.

Mammography does not prevent breast cancer but it helps find tumors in an early stage when there is less invasive and more effective treatment.

The main advantage is the reduction of mortality due to breast cancer for women who participate in the organized screening program.

The principal disadvantage is harder to grasp. Sometimes there is unnecessary and useless treatment (overtreatment), and if it is useless it is harmful, of tumors discovered by the screening that would resolve themselves spontaneously during the woman’s life (overdiagnosis). These malignancies look just like other tumors but either do not grow or grow very slowly. Unfortunately, so far there is no way to distinguish whether a tumor is actually not harmful, so all cases are treated and the woman will not know what her situation was.

It is only possible to estimate the reduction of mortality due to breast cancer by the mammography screening program, and of extra tumor diagnosis (overdiagnosis).

Participation in the screening provides the following benefits and harms to be assessed before taking a decision:

- Reduction of mortality due to breast cancer
- Overdiagnosis
- Less invasive treatments
- False positives or false negatives
- Radiation damage
- Inconvenience of the examination

**What happens in the next 30 years?**


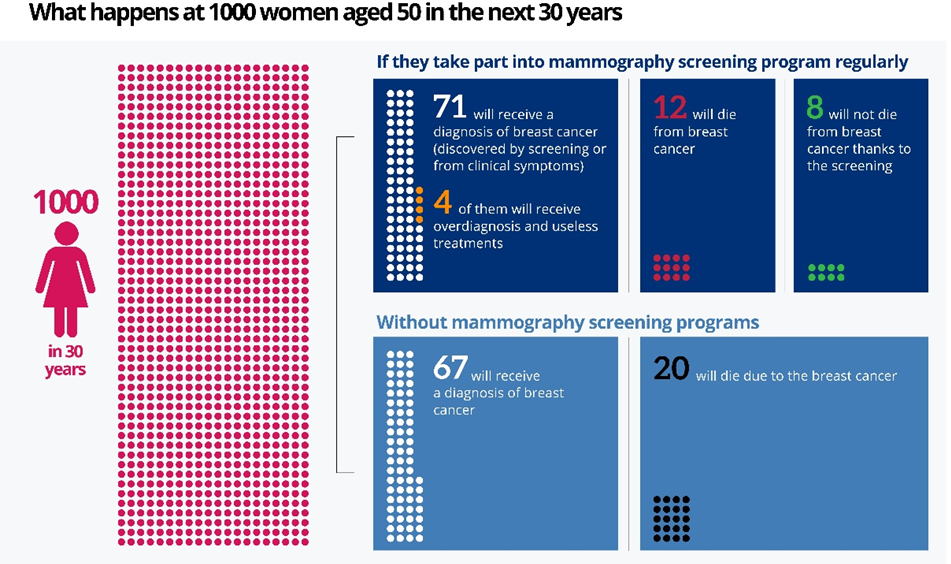


Out of 1000 women aged 50 who regularly participate in a mammography screening program, in the next 30 years:

- 71 will receive a diagnosis of breast cancer (discovered by screening or from clinical symptoms)
- 12 will die from breast cancer
- 8 will not die from breast cancer thanks to the screening
- 4 will receive overdiagnosis and useless treatments

If the same 1000 women are followed without a mammography screening program:

- 67 will receive a diagnosis of breast cancer
- 20 of them will die due to the breast cancer

In other words, in the next 30 years:

Some women will die from breast cancer anyway: 12 of the 1000 who participate in the mammography screening program compared to 20 if the screening program did not exist. Therefore 8 out of 1000 women are saved from death due to breast cancer; 4 of the 1000 women receive a diagnosis and are then treated uselessly for tumors detected by the screening that would probably never emerge. If there is no mammography screening program, there is no overdiagnosis and only evident tumors are treated.

**At what age is mammography screening recommended?**

In Italy the mammography screening program is carried out every two years for women aged 50-69 because there is impressive evidence of the reduction of mortality.

In some Italian regions (Emilia Romagna, Piedmont and Tuscany), the mammography screening program is extended to women aged from 45 to 49 every year and between 70 and 74 every two years. For these age bands the demonstration of the utility of screening is considered sufficient, though the balance of the related benefits and harms is still debated.

The European Code against Cancer (2015) confirmed the recommendation for women aged 50-69 to do the organized mammography screening and, in certain circumstances, also women aged 45-49 and 70-74.

**The risks related to radiation**

Mammography uses X-rays, namely high-energy radiation (also called ionizing) that may damage cells that absorb it, including the development of tumors. Mammography based on quality criteria employs low doses of X-rays so the risk of tumors developing is practically zero.

The amount of radiation from a mammography is comparable to that absorbed in a few weeks from “background radiation”, meaning from radioactive substances in the ground and buildings and to which everyone is normally exposed.

Mammography is comparable to a chest X-ray and is much less dangerous than an abdominal tomography scan.

**Comparison of doses of radiation from different examinations in adults**

| ***Method*** | ***Effective dose in adults**** | ***Time needed to absorb the same dose from background radiation*** |
| --- | --- | --- |
| *Computerized*  *Bone Mineralometry* | *0.001 mSv* | *3 hours* |
| *Limb X-ray* | *0.001 mSv* | *3 hours* |
| *Intraoral X-ray* | *0.005 mSv* | *1 day* |
| *Chest X-ray* | *0.1 mSv* | *10 days* |
| ***Mammography*** | *0.4 mSv* | *7 weeks* |
| *Spinal X-ray* | *1.5 mSv* | *6 months* |
| *Upper abdomen X-ray* | *6 mSv* | *2 years* |
| *Chest computed axial tomography (CAT scan)* | *7 mSv* | *More than 2 years* |
| *Lower abdomen X-ray* | *8 mSv* | *About 3 years* |
| *Abdomen-pelvi CAT scan* | *10 mSv* | *More than 3 years* |

* The effective dose used for the comparison is quantified considering the type of radiation and the specific sensitivity of the body part involved; the measurement units are milliSievert (mSv).

**Organized mammography screening program, a quality program**

In the organized mammography screening program, all women of your age are regularly invited for a free mammography at a clinical center involved in the program that guarantees full assistance, for diagnosis and treatment of any breast cancer.

The quality of all Italian organized mammography screening programs, including the one you are invited to, is monitored and evaluated within national and international initiatives (see the sources above and on the right). Outside this program there is no way of judging, as there is no regular data collection.

At your appointment, a radiographer will take two X-rays for each breast from different angles. The breast is pressed between two plastic plates and this may cause slight discomfort. However, the stronger compression the less radiation you will receive and the more accurate will be the exam.

The X-rays are then assessed by two expert medical radiologists who read at least 5000 exams a year as a quality standard.

If all is well, you will receive a letter or an e-mail. If there is any doubt you will be contacted by phone.

**What result will the mammography give?**

For most women, the result is normal, meaning nothing has been found (negative result). A negative result is reassuring but it does not mean no tumor will ever develop.

Rarely, the tumor can escape observation, (called “false negatives”), for example in hard-to-interpret cases, namely if the breast is dense, or it might develop between this screening mammography and the next invitation: these are referred to as “interval tumors”. Therefore, talk to your doctor if you notice any changes in your breast between two exams, such as nodules you can feel, skin deformation or hardening, or a bleeding or retracting nipple.

A doubtful result or “positive mammography” means the radiologist has seen images like nodules, masses, calcifications or others that could signal a tumor and thus call for more investigation. This may cause you anxiety and concern but it does not imply a malignant disease. If the suspicion is not confirmed by further tests it is called a “false positive”.

**What happens at each screening?**


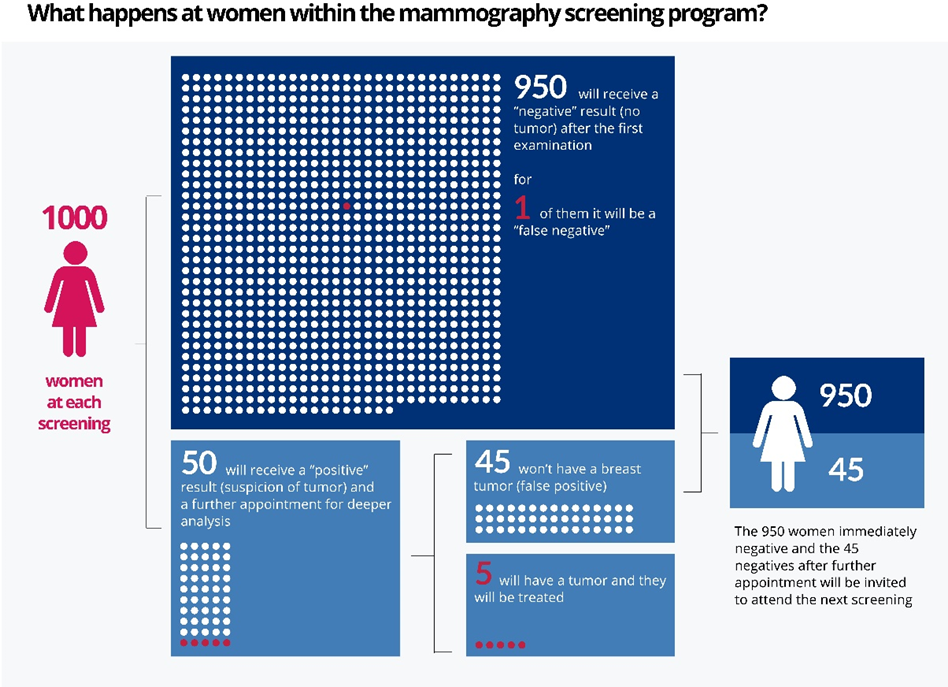


Data from the Italian organized mammography screening program

In Italy, out of 1000 women participating in each screening, on average:

- 950 women will receive a “negative” result (no tumor) after the first examination and will be invited to attend the next examination; for 1-2 of them it will prove to be a “false negative”;
- 50 women of the 1000 will receive a “positive” result (suspicion of tumor) and a further appointment will be set for deeper analysis.

Among these 50 women:

- for 45 of them, the suspicion of a tumor will prove unfounded (false positive) and they will be invited to attend the next screening;
- for the other 5 the suspicion will be confirmed and they will be treated in qualified centers.

In other words:

- In the majority of cases the mammography is normal (negative results for 950 out of 1000 women);
- Many doubtful cases prove normal after further analysis (false positive for 45 out of 50 women);
- There is the extremely rare possibility that a tumor is not identified (false negative). This may happen to 1-2 women out of 1000 and the tumor is generally discovered because of a nodule or breast lesion.

To discover what you can expect 30 years after taking part in a screening program, click here.

**Diagnostic programs in uncertain cases**

When the first mammography gives a doubtful result, the woman is invited for a breast examination and a second mammography and/or echography (both not invasive) to confirm or exclude the real presence of a tumor.

The breast examination is done by a skilled physician who will recognize visually and by palpation even small but significant breast changes.

When it is not possible to exclude a tumor even after further examinations, sampling the suspect nodule is generally suggested (biopsy or fine-needle aspiration): this occurs in about 30% of cases. The result of the biopsy is communicated by the physician who first visited the patient, at her next appointment.

**Breast density**

The breast comprises a glandular part and an adipose one (fat). When the adipose part is the main one the mammography profile is clear, while the appearance is denser when the glandular component is greater.

When the breast is dense the mammography is harder to interpret and a very small tumor may not be detected giving a “false negative” result.

Usually the density changes over time as the glandular part decreases with age. That explains why in general mammography is clearer in older women.

**What is breast cancer and how can it be treated?**

The risk of breast cancer depends on different factors (such as age, family history and life-style) and it is the female tumor that causes the most deaths (see chart 1 *I numeri del cancro in Italia. Rapporto AIOM AIRTUM, 2016).* Nowadays, there is every chance of a cure (see chart 2 *I numeri del cancro in Italia. Rapporto AIOM AIRTUM, 2016)* through the risk of recurrence remains for many years. The probability of a cure depends on the tumor’s biological features.

A tumor can appear in several forms and is caused by uncontrolled growth of breast cells that become malignant.

The tumor cells can grow (when inside the mammary gland this is defined as an *in situ tumor*) and migrate to other organs, in the form of metastasis. A metastatic tumor is harder to fight so prompt intervention is always important.

If the tumor is diagnosed at an early stage, usually treatment is less aggressive.

For most women with breast cancer only the tumor is removed, surgically, with its surrounding tissue, or the whole breast, depending on the extent of local disease. Other treatments, in addition and in different sequences, include radiotherapy, chemotherapy or hormone therapy, which are useful to reduce the chances of the disease recurring. Further therapies are possible or are being tested, especially with biologic drugs for specific groups of patients.

**The main risk and protective factors**

The risk of breast cancer depends on several factors that can be divided into two groups: those that are unchangeable and those that can be reduced by adopting a correct life-style.

**Age**: the risk rises with age and more than 75% of breast cancers are in women over 50.

**Family history**: some families report several cases of breast cancer among first-degree (mother-daughter) or second-degree relations (sisters).

**Genes**: BRCA1 and BRCA2 genetic mutations are responsible for almost half of hereditary breast cancers (5-7% of the total).

Age, family history and genetic patterns are factors to be considered in assessing one’s own risk, though they cannot be change. In addition, other factors can influence the risk of breast cancer, raising or lowering it. For example, a high level of estrogen - the main female hormone - facilitates breast cancer. Any other factors that boost their expression, such as hormone replacement therapy (HRT) increase the risks. Pregnancies lower estrogens production, with a protective effect.

In general, a healthy life-style with a diet rich in fruit and vegetables (unrefined cereals, legumes, non-starchy vegetables, and fruit), less alcohol (no or only one glass of wine a day), weight control, especially after the menopause, can all be protective against breast cancer.

**To reduce the probability of breast cancer**

| ***What to do*** | ***What not to do*** |
| --- | --- |
| *Keep ideal weight (BMI <24,9 kg/m2) with a diet based on vegetables (unrefined cereals, legumes, starchy vegetables, and fruits), no alcohol or 1 glass of wine a day at the most* | *Marked overweight (BMI>28) in menopause* |
| *Follow the Mediterranean diet with a supplement of extra-virgin olive oil* | *Suffer of metabolic syndrome after menopause* |
| *Eat fruit and vegetable during adolescence (≥3 portions a day)* | *Drink more than a single dose of alcohol a day (most risk during adolescence)* |
| *Physical activity (150 minutes of moderate intensity a week at least )* | *Eat large amounts of red meat (≥3 a week)* |
| *Eat fiber (around 30g a day benefits is greatest especially during adolescence)* | *Smoke (most risk during adolescence)* |
| *Follow a vegan diet though it is considered an incomplete diet* | *Use HRT (hormone replacement therapy) in menopause* |
| *Breastfeeding* |  |

**Differences between false positives and overdiagnosis**

It is important to explain the differences between false positives and overdiagnosis.

A “positive result” is considered false when further examinations after mammography exclude breast cancer. “Overdiagnosis” instead means that there is a tumor, it is malignant but will not show itself in the rest of life. Unfortunately, it is still not possible to distinguish a tumor of this type so all are treated and the woman will never know what her situation really was.

**The balance between benefits and harms**

In medicine and healthcare a balance must be drawn up between positive effects (benefits) and negative ones (harm) to decide on the utility of an intervention. For mammography screening, the main benefit is the reduction of mortality due to breast cancer in the long-term, while the main harm is the risk of useless treatments.

Once the main results have been established, a balance must be set to gain an idea of their numbers: how many deaths due to breast cancer are avoided by screening programs? How much overdiagnosis is there, namely cases diagnosed in excess?

When the balance is positive, the health authorities can organize a screening program. Leading scientific authorities and societies, following different strategies, sometimes reach different conclusions on the benefits and especially on the harms.

| ***How many fewer women die of breast cancer thanks to screening?*** | ***How many excess tumors are diagnosed by the screening?*** |
| --- | --- |
| *The mortality reduction found by researchers is between 20% and 38%. The difference depends on the methods used to calculate these estimates, and no single one is universally considered better than another* | *Overdiagnosis ranges from 5% to 30%. The difference depends on the methods used to calculate these estimates, and no single one is universally considered better than another* |
| *How experts have calculated the mortality reduction due to breast cancer* | *How experts have calculated overdiagnosis* |

**How are the rates of specific mortality reduction and overdiagnosis measured?**

Current medical science bases its knowledge on the results of experimental or observational studies, on samples of the subset population. This rigorous method reduces the differences in opinion among physicians but does not eliminate them. The studies cannot give exact figures, but only a range of estimates that are probably close to the real figure (a bit like poll results for elections).

For mammography screening too, experimental and observational studies are analyzed. Experimental ones follow women for some time, dividing them into two groups: those who are take part in the periodical screening program; those who are not invited serve for “comparison”. Each woman is assigned by chance, as if by tossing a coin, to one group or the other. Later, mortality rates and breast cancer frequency in both groups are measured and compared. Experimental studies about mammography screening (8 clinical trials between 1963 and 1991) have evaluated around 500,000 women aged 40-74 in Europe and North America. The observational studies we consider examined screening programs in the 2000s and refer to women included in the screening programs organized according to the European guidelines who received the first invitation for screening, most of them aged 50 and 74 years.

To reach the most reliable conclusion, decisions are based on the results of many studies, called systematic reviews, that analyze together the results of experimental or observational studies, even if they have some different features. A method to decide what type of studies to include (for example only experimental, only observational, or both) is needed in order to weigh their different quality.

Screening programs started on the basis of experimental study results, in the early 1990s. The age for starting screening was debated: 40 years in the USA and more than 50 in Europe (usually up to 69). After that, several research groups conducted systematic reviews of all the studies using methods and strategies for quality assessment that have given different results, leading to debate among the researchers themselves.

For further reviews of results about mortality rates click here; for those about overdiagnosis click here.

**Different estimates of the reduction of mortality due to breast cancer**

The main reviews considered are the recent ones by the Cochrane collaboration (systematic review of experimental studies updated to 2013) and the Independent UK Panel (2012) that also evaluated only experimental studies; the Euroscreen Group included only observational studies based on European programs (2012) (see the sources).

The Cochrane review of experimental studies considers all invited women aged 40-74 and followed for 13 years, and estimates around a reduction of 20% of specific mortality for the whole series of studies, and around 0% omitting the less reliable studies.

The Independent UK Panel review, based on the same experimental studies and on all invited women aged 40-74 and followed for 13 years, also found a 20% mortality reduction, considering all the sufficiently reliable studies.

The Euroscreen review of European observational studies estimated a reduction of mortality due to breast cancer around 25% for women aged 50-69 in an organized mammography screening program. In addition, the reduction of mortality due to breast cancer for women regularly participating in a mammography screening program was about 38%.

Both the Cochrane and the UK Panel estimates are based on the same studies but used different methods and definitions. Those used by the Euroscreen group were similar to those used by the UK Panel even though the former examined observational studies, so the three reviews are not directly comparable. The Euroscreen appraisal answers to the question of what a woman would expect on deciding to take part in a European screening program, concerning the reduction of specific mortality. Therefore, the planners of the screening program you are invited to attend consider the Euroscreen estimates as most reliable to inform individual choices.

**Different overdiagnosis estimates**

The main reviews considered are the recent ones by the Cochrane collaboration (systematic review of experimental studies updated to 2013) and the Independent UK Panel (2012) that also evaluated only experimental studies; the Euroscreen Group included only observational studies based on European programs (2012) (see the sources).

To quantify overdiagnosis:

- From a general population perspective, the question is: “Out of all the women aged between 50 and 80 years who receive a diagnosis of breast cancer, what is the proportion overdiagnosed by the screening?”. To answer, the Euroscreen review in 2012, that included only observational studies, estimated that out of 1000 women aged 50 starting with a regular screening program, over the next 30 years 71 cancers will be found and 4 of them will be overdiagnosed and treated uselessly. That harm is around 5% in relative terms (measure A);
- From an individual perspective, the question is: “Participating in a screening program and receiving a diagnosis of tumor by the screening, what is the probability that it is an overdiagnosis?”. The estimate is around 10% (measure B)

The difference is due in particular to the fact that measure B considers only women diagnosed due to the screening.

In the Independent UK Panel’s review, based on three experimental studies, overdiagnosis reached about 11% when expressed as the proportion of all tumors found among the invited women (measure A), and 19% as the proportion of only tumors diagnosed during the active screening period (measure B).

The Cochrane review, based on several experimental studies (ages 40-74), analyzed the excess of treatments (mastectomies and conservative procedures), indirectly reaching an estimate of overdiagnosis around 30%, including - as the denominator - all tumors diagnosed in the control group at the end of the follow-up.

The Cochrane and UK Panel’s estimates are both based on experimental studies but used different methods and definitions. Those used by the Euroscreen group were similar to those used by the UK Panel but analyzed observational studies, so the three reviews are not directly comparable.

### **Who we are**

### This project is coordinated by IRCCS Istituto di Ricerche Farmacologiche Mario Negri in collaboration with Lega Italiana Lotta contro i Tumori-Firenze, Zadig Agenzia di Editoria Scientifica, GISMa Gruppo Italiano Screening Mammografico and with Prevenzione Serena di Torino, dell’Unità Operativa Centro Gestionale Screening di Palermo e dell’Istituto per lo Studio e la Prevenzione Oncologica di Firenze.

### The project is funded by AIRC, the Italian Association for Cancer Research- IG2015-17274.

- Istituto di Ricerche Farmacologiche Mario Negri IRCCS: Paola Mosconi, Anna Roberto, Cinzia Colombo
- Zadig Agenzia di Editoria Scientifica: Giulia Candiani, Roberto Satolli
- Lega Italiana Lotta contro i Tumori – Firenze: Eugenio Paci
- GISMa Gruppo Italiano Screening Mammografico e Prevenzione Serena – Torino: Livia Giordano
- Istituto per lo Studio, Prevenzione e la Rete Oncologica Firenze: Paola Mantellini
- Unità Operativa Centro Gestionale Screening di Palermo: Mario Valenza
- Centro Screening Oncologici di Reggio Emilia: Cinzia Campari
- SSD Unità di Valutazione e Organizzazione Screening, Dipartimento di Prevenzione – Cuneo: Lorenzo Marcello Orione
- UOC Medicina Preventiva delle Comunità – Screening, UOS di Milano: Silvia De Andrea

**Value clarification exercise**

To make a choice you need correct and complete information. Here is a map of all the contents, with the pages you have already visited highlighted.

However, we realise that your choice will not be based only on the information acquired, and other points will influence the decision: your values, your experience, your perception of the risk of developing this disease. These are important aspects that guide many of our choices and this is why we have returned to them in this final section.

Here are the main points that can influence your decision to participate in mammography screening. For each one you will find a cursor that you can move for or against participation or that you can keep in the middle if that particular aspect doesn't matter to you.

The whole picture which you can print and share with people you trust - gives an overview of your position.

1. **The reduction in breast cancer mortality thanks to early diagnosis motivates you…**

-3 -2 -1 0 1 2 3

Against participation Doesn't count In favor of participation

1. **Breast conservation thanks to early diagnosis motivates you…**

-3 -2 -1 0 1 2 3

Against participation Doesn't count In favor of participation

1. **Your perception of the risk of developing a tumor motivates you…**

-3 -2 -1 0 1 2 3

Against participation Doesn't count In favor of participation

1. **The quality of the organized screening program offered by the public health service motivates you…**

-3 -2 -1 0 1 2 3

Against participation Doesn't count In favor of participation

1. **The risk of unnecessary treatments (overdiagnosis and overtreatment) motivates you…**

-3 -2 -1 0 1 2 3

Against participation Doesn't count In favor of participation

1. **The fear of a diagnosis of breast cancer and its consequences motivates you…**

-3 -2 -1 0 1 2 3

Against participation Doesn't count In favor of participation

1. **The anxiety of the examination and having to wait for the result motivates you…**

-3 -2 -1 0 1 2 3

Against participation Doesn't count In favor of participation

1. **The harm of radiation received with mammography motivates you…**

-3 -2 -1 0 1 2 3

Against participation Doesn't count In favor of participation

1. **The possibility of a false positive result, that meaning the suspicion of a tumor that is not actually there motivates you…**

-3 -2 -1 0 1 2 3

Against participation Doesn't count In favor of participation

1. **The fact that experts disagree on the size of the main benefits and harms for you motivates you….**

-3 -2 -1 0 1 2 3

Against participation Doesn't count In favor of participation

**Supplementary 3. Questionnaires**

**BASELINE QUESTIONNAIRE**

**SOCIODEMOGRAFIC**

Nationality:

a) Italian

b) Other, please specify_________________________

Education

□ Elementary

□ Lower middle

□ Higher middle

□ Degree

□ Other

Marital status

□ Single

□ Married or living together

□ Separated or divorced

□ Widowed

Employment status

□ Paid work (full or part time)

□ No paid work (retired, housewife, other)

Do you use internet to search for health information?

□ Never

□ A few times a month

□ At least once a week

□ Several times a week

□ Daily

Have you already had a mammography?

□ Yes, in a public facility, when? __________(year)

□ Yes, in a private facility, when? __________(year)

□ No

Has anyone in your family or your friends had breast cancer?

□ Yes

□ No

Have you ever had a tumor?

□ Yes If Yes, type of tumor _____________________________________________

□ No

Have you participated in other organized screening programs?

Fecal occult blood tests for colorectal cancer □ Yes □ No

Pap test for cervical cancer □ Yes □ No

**PERCEIVED RISK**

**Perceived risk of breast cancer relative to the average woman**

| Much lower | A bit lower | About the same | A bit higher | Much higher |
| --- | --- | --- | --- | --- |

**KNOWLEDGE**

**What is a SCREENING mammogram?**

□ A mammogram you have when you're healthy

□ A mammogram you have if you notice a change or lump in your breast

**An organized mammography screening program can detect a breast cancer in an early stage and lead to less invasive surgery and treatment**

□ True

□ False

**Which of the following statements about mammography reflects your opinion?**

***Regular mammography every two years in women who are well*:**

□ Prevents the risk of breast cancer

□ Does not prevents the risk of breast cancer

**Who do you think is more likely to die from breast cancer?**

□ Women who have screening mammograms

□ Women who do not have screening mammograms

**Do you think a screening mammogram will find every breast cancer?**

□ Yes

□ No

**Do all women with an abnormal screening mammogram result have breast cancer?**

□ Yes

□ No

**Which of these 2 statements best describes over-detection?**

□ Screening finds a cancer that would never have caused trouble

□ Screening finds an abnormality but extra tests show it is not cancer

**Screening leads some women with a harmless cancer to get treatment they do not need.**

□ True

□ False

**In the organized mammography screening program, the presence of two expert radiologists increases the ability to identify a breast tumor**

□ True

□ False

**The usefulness of an organized mammography screening program is:**

□ Totally recognized by doctors and researchers

□ Questioned by some doctors and researchers

**For the next few questions, I would like you to imagine 1000 ordinary women who are 50 years old who have participated regularly in organized mammography screening program for 30 years**

**How many women do you think will avoid dying from breast cancer because of screening?**

□ 8

□ 50

□ 150

**How many women do you think will be diagnosed and treated for a breast cancer that is not harmful?**

□ 4

□ 25

□ 70

**Now, I would like you to imagine 1000 ordinary women who are 50 years old who have not participated in organized mammography screening program, in their next 30 years…. How many die of breast cancer?**

□ 20

□ 80

□ 140

**ATTITUDE**

**For you, having breast screening is…..**

A bad thing Not a bad thing

1 2 3 4 5

Beneficial Not beneficial

1 2 3 4 5

Harmful Not harmful

1 2 3 4 5

A good thing Not a good thing

1 2 3 4 5

Worthwhile Not worthwhile

1 2 3 4 5

Important Unimportant

1 2 3 4 5

**INTENTION**

**Intending to be screened**

| Definitely will | Likely to | Unsure | Not likely to | Definitely will not |
| --- | --- | --- | --- | --- |

**FOLLOW-UP QUESTIONNAIRE**

**ATTITUDE**

**For you, having breast screening is…..**

A bad thing Not a bad thing

1 2 3 4 5

Beneficial Not beneficial

1 2 3 4 5

Harmful Not harmful

1 2 3 4 5

A good thing Not a good thing

1 2 3 4 5

Worthwhile Not worthwhile

1 2 3 4 5

Important Unimportant

1 2 3 4 5

**SATISFACTION/ACCEPTABILITY OF THE INFORMATION**

| **Thinking back to the type of information material you have read ...** | | | |
| --- | --- | --- | --- |
| Was there enough information? | Too much | Too little | Fair |
| Was the information on benefit new to you? | All or almost all | Some | None |
| Was the information on harm new to you? | All or almost all | Some | None |
| Was the information clear? | All or almost all | Some | None |
| The information seemed… | In favor of screening | Balanced | Against screening |
| Didi t help you to decide? | Yes | Not much | No |
| Would you recommend it to other women? | Yes | Not much | No |
| Only for Decision-Aid  Was the controversy new to you? | All or almost all | Some | None |

**DECISIONAL CONFLICT (SURE QUESTIONNAIRE)**

**Sure of myself**

**Do you feel SURE about the best choice for you?**

□ Yes

□ No

**Understanding information**

**Do you know the benefits and risks of each option?**

□ Yes

□ No

**Risk-benefit ratio**

**Are you clear about which benefits and risks matter most to you?**

□ Yes

□ No

**Encouragement**

**Do you have enough support and advice to make a choice?**

□ Yes

□ No

**INTENTION**

**Intending to be screened**

| Definitely will | Likely to | Unsure | Not likely to | Definitely will not |
| --- | --- | --- | --- | --- |

**KNOWLEDGE**

**What is a SCREENING mammogram?**

□ A mammogram you have when you're healthy

□ A mammogram you have if you notice a change or lump in your breast

**An organized mammography screening program can detect a breast cancer in an early stage and lead to less invasive surgery and treatment**

□ True

□ False

**Which of the following statements about mammography reflects your opinion?**

***Regular mammography every two years in women who are well*:**

□ Prevents the risk of breast cancer

□ Does not prevents the risk of breast cancer

**Who do you think is more likely to die from breast cancer?**

□ Women who have screening mammograms

□ Women who do not have screening mammograms

**Do you think a screening mammogram will find every breast cancer?**

□ Yes

□ No

**Do all women with an abnormal screening mammogram result have breast cancer?**

□ Yes

□ No

**Which of these 2 statements best describes over-detection?**

□ Screening finds a cancer that would never have caused trouble

□ Screening finds an abnormality but extra tests show it is not cancer

**Screening leads some women with a harmless cancer to get treatment they do not need.**

□ True

□ False

**In the organized mammography screening program, the presence of two expert radiologists increases the ability to identify a breast tumor**

□ True

□ False

**The usefulness of an organized mammography screening program is:**

□ Totally recognized by doctors and researchers

□ Questioned by some doctors and researchers

**For the next few questions, I would like you to imagine 1000 ordinary women who are 50 years old who have participated regularly in organized mammography screening program for 30 years**

**How many women do you think will avoid dying from breast cancer because of screening?**

□ 8

□ 50

□ 150

**How many women do you think will be diagnosed and treated for a breast cancer that is not harmful?**

□ 4

□ 25

□ 70

**Now, I would like you to imagine 1000 ordinary women who are 50 years old who have not participated in organized mammography screening program, in their next 30 years…. How many die of breast cancer?**

□ 20

□ 80

□ 140

**Supplementary 4. Figure 1. Value clarification exercise**


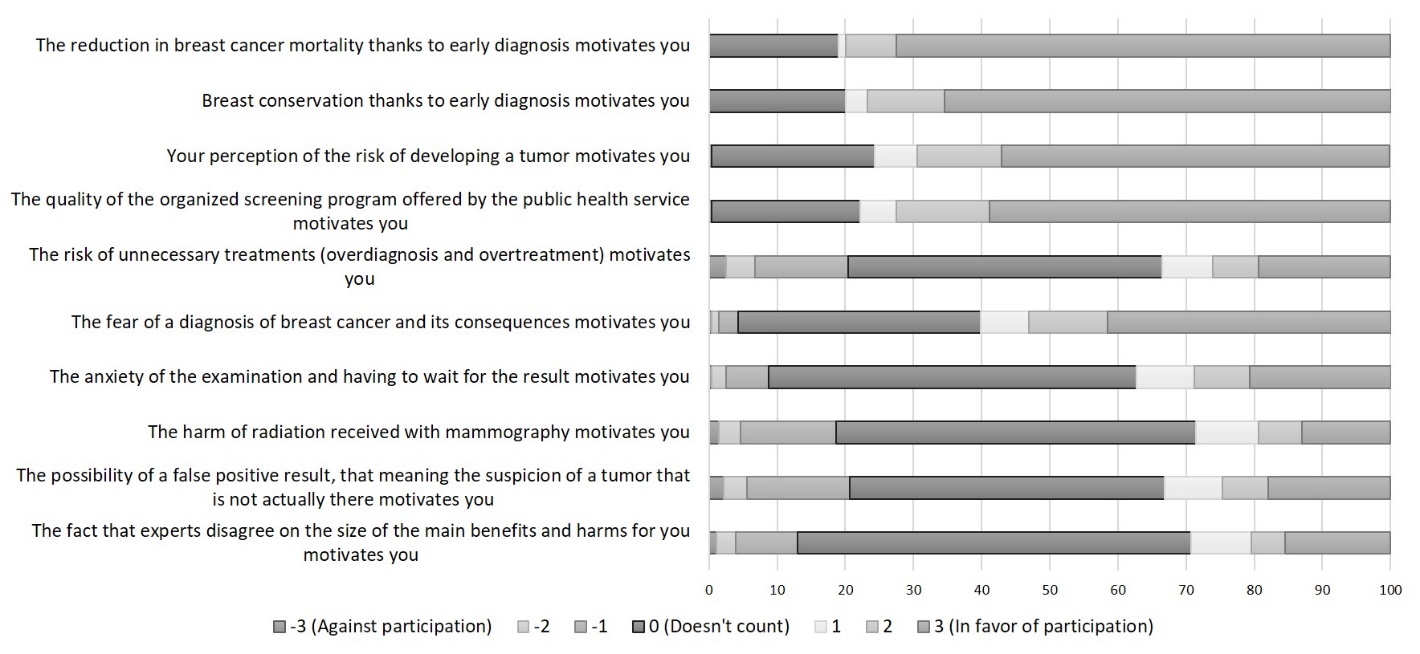

Supplement: Supplementary file 1 — Supplementary files [file 41416_2020_935_MOESM1_ESM.docx]
